# Ultrafast Fabrication of H_2_SO_4_, LiCl, and Li_2_SO_4_ Gel Electrolyte Supercapacitors with Reduced Graphene Oxide (rGO)-LiMnO_x_ Electrodes Processed Using Atmospheric-Pressure Plasma Jet

**DOI:** 10.3390/mi14091701

**Published:** 2023-08-30

**Authors:** Pei-Ling Lan, I-Chih Ni, Chih-I Wu, Cheng-Che Hsu, I-Chun Cheng, Jian-Zhang Chen

**Affiliations:** 1 Graduate Institute of Applied Mechanics, National Taiwan University, Taipei City 10617, Taiwan; r11543037@ntu.edu.tw; 2 Advanced Research Center for Green Materials Science and Technology, National Taiwan University, Taipei City 10617, Taiwan; 3 Department of Electrical Engineering, Graduate Institute of Photonics and Optoelectronics, National Taiwan University, Taipei City 10617, Taiwan; ichihni@ntu.edu.tw (I.-C.N.); chihiwu@ntu.edu.tw (C.-I.W.); iccheng@ntu.edu.tw (I.-C.C.); 4 Graduate School of Advanced Technology, National Taiwan University, Taipei City 10617, Taiwan; 5 Department of Chemical Engineering, National Taiwan University, Taipei City 10617, Taiwan; chsu@ntu.edu.tw

**Keywords:** supercapacitor (SC), atmospheric-pressure plasma (APP), flexible electronics, reduced graphene oxide (rGO)

## Abstract

Pastes containing reduced graphene oxide (rGO) and LiCl-Mn(NO_3_)_2_·4H_2_O are screen-printed on a carbon cloth substrate and then calcined using a nitrogen atmospheric-pressure plasma jet (APPJ) for conversion into rGO-LiMnO_x_ nanocomposites. The APPJ processing time is within 300 s. RGO-LiMnO_x_ on carbon cloth is used to sandwich H_2_SO_4_, LiCl, or Li_2_SO_4_ gel electrolytes to form hybrid supercapacitors (HSCs). The areal capacitance, energy density, and cycling stability of the HSCs are evaluated using electrochemical measurement. The HSC utilizing the Li_2_SO_4_ gel electrolyte exhibits enhanced electrode–electrolyte interface reactions and increased effective surface area due to its high pseudocapacitance (PC) ratio and lithium ion migration rate. As a result, it demonstrates the highest areal capacitance and energy density. The coupling of charges generated by embedded lithium ions with the electric double-layer capacitance (EDLC) further contributed to the significant overall capacitance enhancement. Conversely, the HSC with the H_2_SO_4_ gel electrolyte exhibits better cycling stability. Our findings shed light on the interplay between gel electrolytes and electrode materials, offering insights into the design and optimization of high-performance HSCs.

## 1. Introduction

Atmospheric-pressure plasmas (APPs) are a favorable alternative to vacuum plasmas as they do not require an expensive vacuum pump and chamber. As an APP is not limited by the dimensions of a vacuum chamber, it enables flexible processing with various substrate sizes [1]. APPs can be used for various applications including surface cleaning, altering surface physical and chemical properties, modifying surface topography, and depositing materials [2,3]. A nitrogen APP can be used for the nitrogen doping of materials [4,5].

Supercapacitors (SCs) have attracted much interest because they afford advantages including high power density, rapid charging and discharging rates, and exceptional cycling stability [6]. In SCs, electric double-layer capacitance (EDLC) and pseudocapacitance (PC) can be used as energy storage mechanisms. An SC in which both EDLC and PC mechanisms are used simultaneously is called a hybrid supercapacitor (HSC) [7,8,9]. Flexible HSCs can be fabricated through using flexible substrates and flexible electrode materials. Flexible HSCs can be applied in fields such as wearable devices and foldable displays, where they provide greater freedom and flexibility in the manufacturing and integration of electronic devices [10,11,12]. Graphene is a two-dimensional material with exceptional properties; therefore, graphene plays a crucial role in fields such as supercapacitors and multiple plasmon-induced transparency metasurfaces, highlighting its multifaceted applications [13,14,15,16]. Adding reduced graphene oxide (rGO), a flexible electrode material renowned for its high conductivity and flexibility, can enhance the overall performance of HSCs through providing a porous structure that offers a greater surface area for charge storage [17,18,19,20,21]. Carbon cloth, as a flexible substrate, has a highly porous 3D structure formed by the interlaced arrangement of fibers; this facilitates rapid electron and ion transport for HSC devices. This porous structure provides a larger surface area, enabling more efficient charge storage and release. Additionally, the interwoven fiber arrangement enhances the mechanical strength and flexibility, making carbon cloth an ideal substrate material for fabricating flexible HSCs [22]. Adding lithium ions in the electrolyte can enhance the electrochemical stability and specific capacitance of the HSC, because lithium ions have a higher migration rate and can undergo fast and reversible ion insertion/extraction reactions on the electrode material surface. This enhances the charge storage capacity and cycle life of the HSC [23]. For flexible HSCs, gel electrolytes offer mechanical flexibility with ion transport capability. Additionally, they can reduce or eliminate the risk of electrolyte leakage. The controllable gel state ensures a stable and confined electrolyte system. These factors make gel electrolytes advantageous in specific applications where reliable ion conductivity and minimal leakage risk are desired [24,25,26].

This study focuses on the ultrafast (<300 s) fabrication of HSCs with rGO-LiMnO_x_ nanocomposite electrodes using a nitrogen atmospheric-pressure plasma jet (APPJ). HSCs with three different gel electrolytes, H_2_SO_4_, LiCl, and Li_2_SO_4_, are evaluated and compared. Based on the performance characteristics of HSC materials under different gel electrolyte conditions, we study the compatibility between gel electrolytes and electrode materials, aiming to identify superior material combinations for optimal synergistic effects. This research contributes to the design and optimization of high-performance HSCs.

## 2. Experimental

### 2.1. Preparation of rGO-LiCl-Mn(NO_3_)_2_·4H_2_O Pastes

RGO-LiCl-Mn(NO_3_)_2_·4H_2_O pastes were prepared via mixing 0.05 g of rGO (thickness: <5 nm, sheet size: 0.1–5 μm; Golden Innovation Business Co., Ltd., Taipei City, Taiwan), 0.04 g of LiCl (lithium chloride, anhydrous, 99%, Alfa Aesar, Ward Hill, MA, USA), 0.3 g of Mn(NO_3_)_2_·4H_2_O (manganese (II) nitrated tetrahydrate, 98%, Alfa Aesar, Ward Hill, USA), 3.245 g of terpineol (anhydrous, #86480, Aldrich, Munich, Germany), 1.5 g of ethanol, 1.75 g of ethyl cellulose (#46070, Sigma, Munich, Germany), and 2.25 g of ethyl cellulose (#46080, Sigma, Munich, Germany) [27]. The mixture was stirred at 850 rpm for 24 h using a magnetic stirrer and then condensed using a rotatory evaporator at 55 °C for 6 min to obtain the pastes.

### 2.2. Fabrication of HSCs

RGO-LiCl-Mn(NO_3_)_2_·4H_2_O pastes were screen-printed onto carbon cloth three times, and they finally covered an area of 1.5 cm × 2 cm. After screen-printing, the pastes were dried in an oven at 100 °C for 10 min [28]. Next, the carbon cloth was treated with a nitrogen APPJ for 180 and 300 s. The temperature of the substrate reached approximately 620 °C (nitrogen flow rate = 46 slm) during the APPJ process [4]. The APPJ treatment process burned out the ethyl cellulose and modified the materials in the selected area [29]. After APPJ processing, rGO-LiMnO_x_ nanocomposites were formed on the carbon cloth. Three types of gel electrolytes were used in the HSCs: H_2_SO_4_, LiCl, and Li_2_SO_4_. For the H_2_SO_4_ gel electrolyte, 1.5 g of polyvinyl alcohol (PVA; 99+% hydrolyzed, Aldrich), and 15 mL of 1 M H_2_SO_4_ were mixed using a magnetic stirrer at a rotation speed of 200 rpm in a water bath at 80 °C until the solution became clear without any sediment. Then, the mixture was stirred at room temperature at 850 rpm for 1 h. Similarly, to prepare the LiCl gel electrolyte, 1.5 g of PVA and 15 mL of 1 M LiCl were mixed at 90 °C until the solution became clear, and then, it was stirred at room temperature for 1 h [30]. For the Li_2_SO_4_ gel electrolyte, two solutions were prepared: 1.5 g of PVA and 10 mL of DI water were mixed at 90 °C until the solution became clear, and 3 g of BMIMCl (1-butyl-3-methylimidazolium chloride, 98%, Sigma), 1.65 g of Li_2_SO_4_ (lithium sulfate, anhydrous, 99%, Alfa Aesar), and 5 mL of DI water were mixed at 90 °C until the solution became clear. The two solutions were mixed at 90 °C and then freeze-dried for 24 h [31].

**Figure 1:**
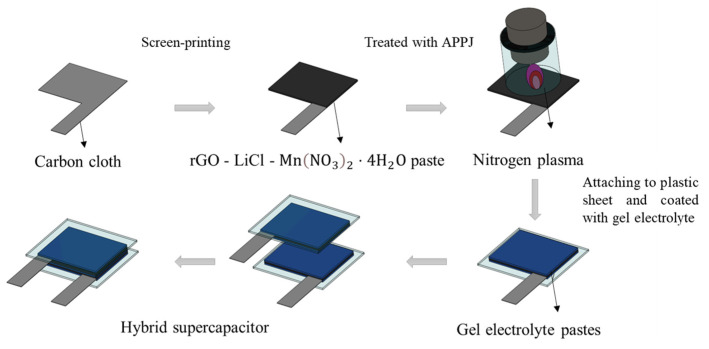
Fabrication process of gel electrolyte HSC.

For HCs with H_2_SO_4_ and LiCl gel electrolytes, 0.5 mL of the gel electrolyte was spread on an rGO-LiMnO_x_ carbon cloth electrode and left to dry at room temperature for 24 h. This process was repeated three times. Finally, two electrodes coated with the gel electrolyte were placed together with the gel sides facing each other to create a sandwich-type HSC. The fabrication process of the gel electrolyte HSCs is shown in Figure 1. For Li_2_SO_4_ gel electrolyte HSCs, the Li_2_SO_4_ gel electrolyte was deposited on an rGO-LiMnO_x_ carbon cloth electrode before freezing it for 24 h. Next, another layer of mixed solution was dropped and covered with the frozen solution. Finally, two pieces of samples were combined and frozen again for another 24 h.

### 2.3. Characterization of rGO-LiMnO_x_ and HSCs

After APPJ treatment, the electrode material transformed into rGO-LiMnO_x_. The structure of rGO-LiMnO_x_ was analyzed using scanning electron microscopy (SEM, JSM-7800F Prime, JEOL, Tokyo, Japan). The water contact angle of rGO-LiMnO_x_ on carbon cloth was measured using a goniometer (Model 100SB, Sindetake, Taipei City, Taiwan). X-ray photoelectron spectroscopy (XPS, Sigma Probe, Thermo VG Scientific, Waltham, MA, USA) analysis was conducted using an Al-Kα source (1486.6 eV) to investigate the surface chemical bonding state.

Cyclic voltammetry (CV; potential window: 0−0.8 V, potential scan speed: 2−200 mV s^−1^), galvanostatic charging/discharging (GCD; potential window: 0−0.8 V, constant current: 4, 2, 1, 0.5, and 0.25 mA), and electrochemical impedance spectroscopy (EIS; 0.1–100,000 Hz) experiments were performed for HSCs with H_2_SO_4_, LiCl, and Li_2_SO_4_ gel electrolytes using an electrochemical workstation (PGSTAT204, Metrohm Autolab, Utrecht, The Netherlands).

## 3. Results and Discussion

### 3.1. SEM Inspection

**Figure 2:**
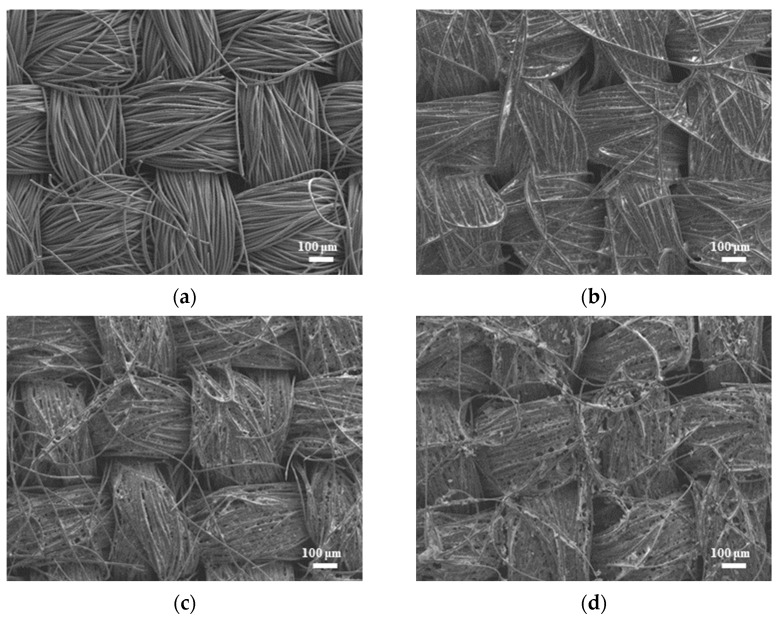
SEM images (magnification: 80×) of (**a**) carbon cloth and (**b**) untreated pastes on carbon cloth and rGO-LiMnO_x_ after APPJ treatment for (**c**) 180 s and (**d**) 300 s.

**Figure 3:**
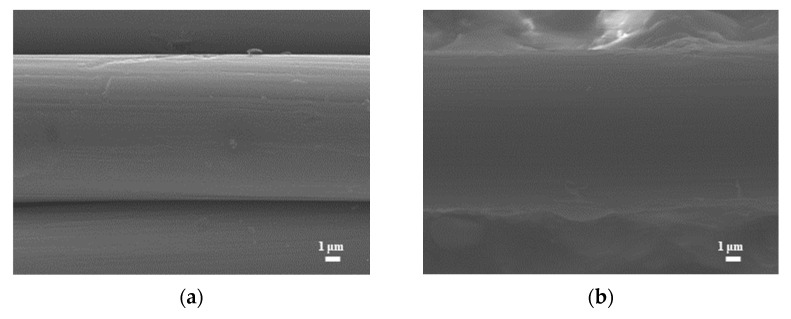

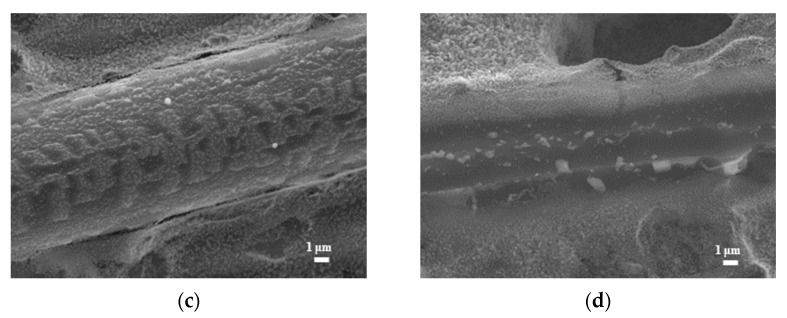
SEM images (magnification: 5000×) of (**a**) carbon cloth and (**b**) untreated pastes on carbon cloth and rGO-LiMnO_x_ after APPJ treatment for (**c**) 180 s and (**d**) 300 s.

**Figure 4:**
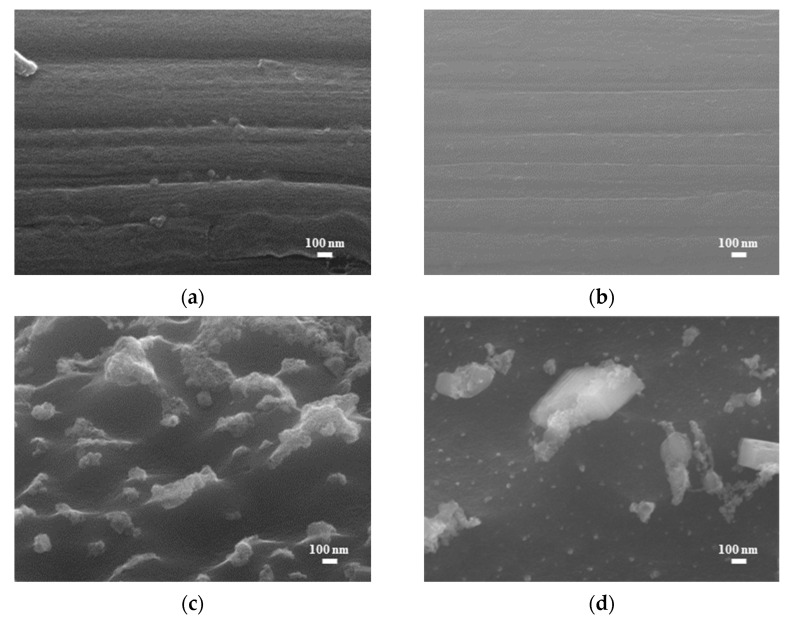
SEM images (magnification: 50,000×) of (**a**) carbon cloth and (**b**) untreated pastes on carbon cloth and rGO-LiMnO_x_ after APPJ treatment for (**c**) 180 s and (**d**) 300 s.

Figure 2 shows SEM images (magnification: 80×) of the bare carbon cloth, untreated pastes, and APPJ-treated pastes. After screen-printing the pastes, the space between the carbon fibers was filled with the pastes. The SEM images in Figure 3 (magnification: 5000×) show that after APPJ treatment, most of the ethyl cellulose was burned off, and the pastes were converted into rGO-LiMnO_x_. The SEM images in Figure 4 (magnification: 50,000×) show that surface particles tend to aggregate after APPJ processing.

### 3.2. Water Contact Angles of rGO-LiMnO_x_

**Figure 5:**
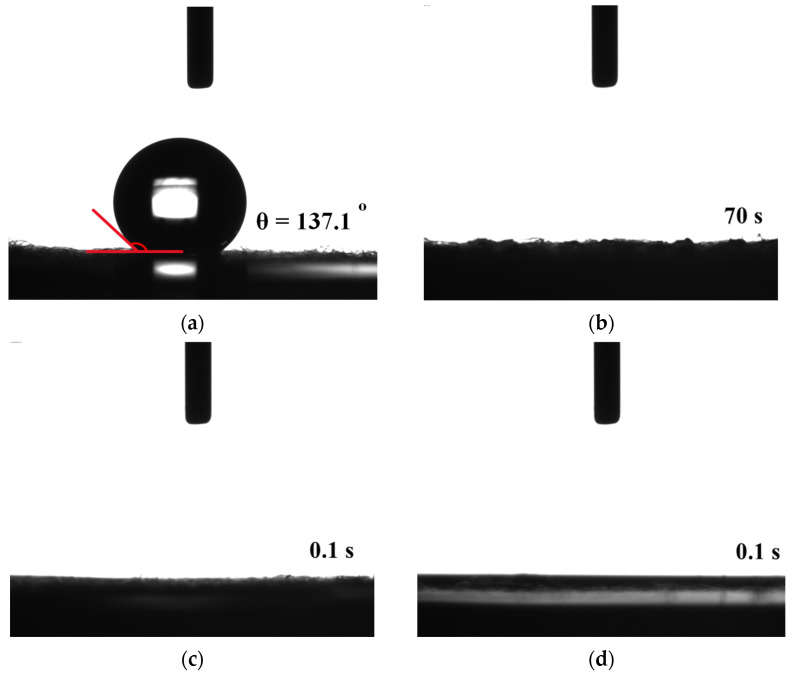
Water contact angles of (**a**) carbon cloth and (**b**) untreated pastes on carbon cloth and after APPJ treatment for (**c**) 180 s and (**d**) 300 s.

Figure 5 shows the water contact angle results for the screen-printed pastes and rGO-LiMnO_x_ after APPJ treatment. The pristine carbon cloth exhibits a high water contact angle of 137.1°, indicating that it is hydrophobic. A previous study suggested that pure rGO exhibits hydrophobic characteristics [32]. In contrast, the as-deposited and APPJ-treated samples exhibited hydrophilic behavior, with water droplets completely penetrating the substrates during testing [33,34]. The difference in hydrophilicity can be discerned through observing the droplet penetration time. For as-deposited pastes on carbon cloth, the droplet takes approximately 70 s to penetrate the substrate; for APPJ-treated samples, the droplet immediately penetrates the substrate. These results indicate that the precursors of lithium manganese oxides are hydrophilic. The reactive plasma species generated by the APPJ can penetrate the porous structure of the carbon cloth, leading to more thorough surface modification. This, in turn, results in the long-lasting hydrophilicity of the carbon cloth [35].

### 3.3. XPS Results of rGO-LiMnO_x_

**Figure 6:**
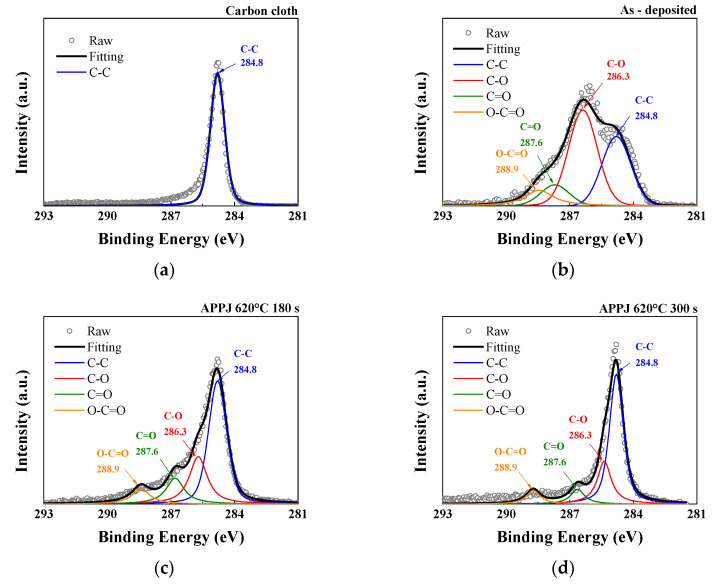
XPS C1s spectra of (**a**) carbon cloth and (**b**) untreated pastes on carbon cloth and APPJ-treated samples for (**c**) 180 s and (**d**) 300 s.

The C1s spectrum can be resolved into four peaks representing different chemical bonds: C–C, C–O, C=O, and O–C=O at binding energies of 284.8, 286.3, 287.6, and 288.9 eV, respectively [36,37]. The analysis of the C1s peak in Figure 6 and Table 1 showed that in addition to the C–C bond of the carbon cloth, the deposited carbon cloth shows peaks related to the C–O, C=O, and O–C=O bonds. The C–O, C=O and O–C=O bonds primarily originate from interactions between the oxide and the substrate. Some oxygen from the environment also participates in the reaction during the APPJ process. Furthermore, after nitrogen APPJ treatment, the oxygen content decreased, especially in the form of the C–O bond, and the main peak reverted to the C–C bond, indicating the presence of ethyl cellulose and the oxidation and evaporation caused by the APPJ treatment [38].

**Figure 7:**
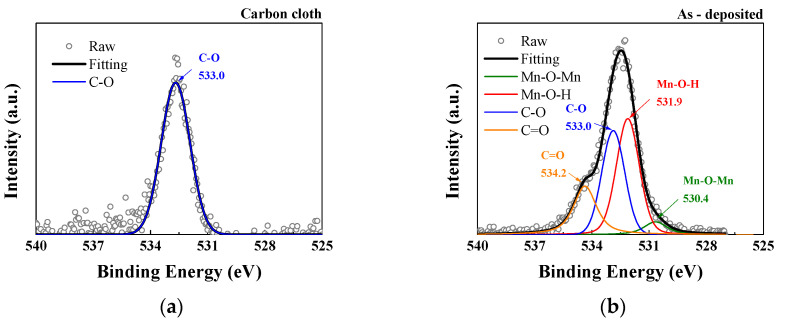

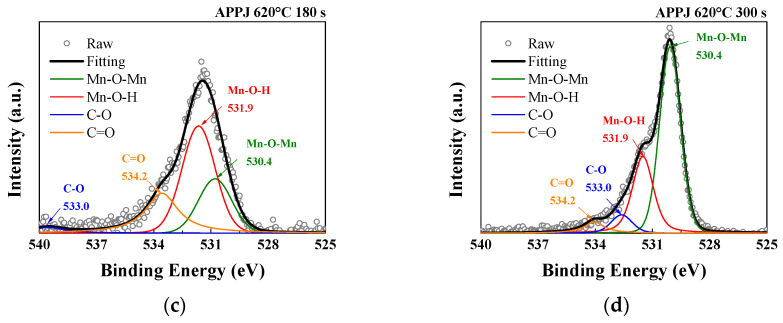
XPS O1s spectra of (**a**) carbon cloth and (**b**) untreated pastes on carbon cloth and APPJ-treated samples for (**c**) 180 s and (**d**) 300 s.

The O1s spectrum can be resolved into four peaks representing different chemical bonds: Mn–O–Mn, Mn–O–H, C–O, and C=O at binding energies of 530.4, 531.9, 533, and 534.2 eV, respectively [39]. In the deposited carbon cloth, the high proportion of C–O bond components is attributed to the surface coverage of ethyl cellulose, similar to the findings in the C1s analysis. At elevated temperature, structural water is released, and the deposited manganese oxide is dehydrated. As shown in Figure 7 and Table 2, as the APPJ treatment temperature increased to 620 °C, the presence of Mn–O–H bonds decreased. This results in a significant reduction in the hydroxide composition, with anhydrous Mn–O–Mn becoming the dominant oxide species [40].

**Figure 8:**
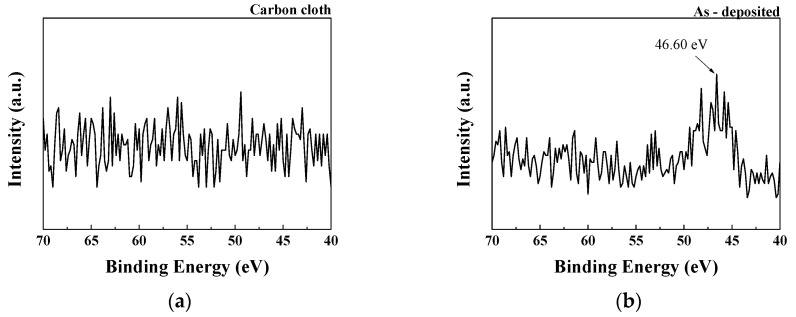

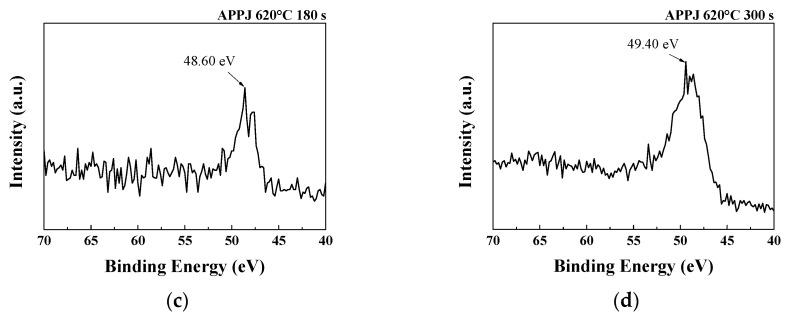
XPS Li1s spectra of (**a**) carbon cloth and (**b**) untreated pastes on carbon cloth and APPJ-treated samples for (**c**) 180 s and (**d**) 300 s.

Figure 8 shows that the paste exhibits obvious Li1s peaks before and after APPJ treatment. After APPJ treatment, the increased binding energy, which is better at a treatment time of 300 s than at that of 180 s, indicates the stronger interaction between Li atoms and the HSC electrode material. This enhanced interaction enables more efficient charge adsorption and storage in the material, thereby increasing the energy density and charge storage capacitance of the HSC.

**Figure 9:**
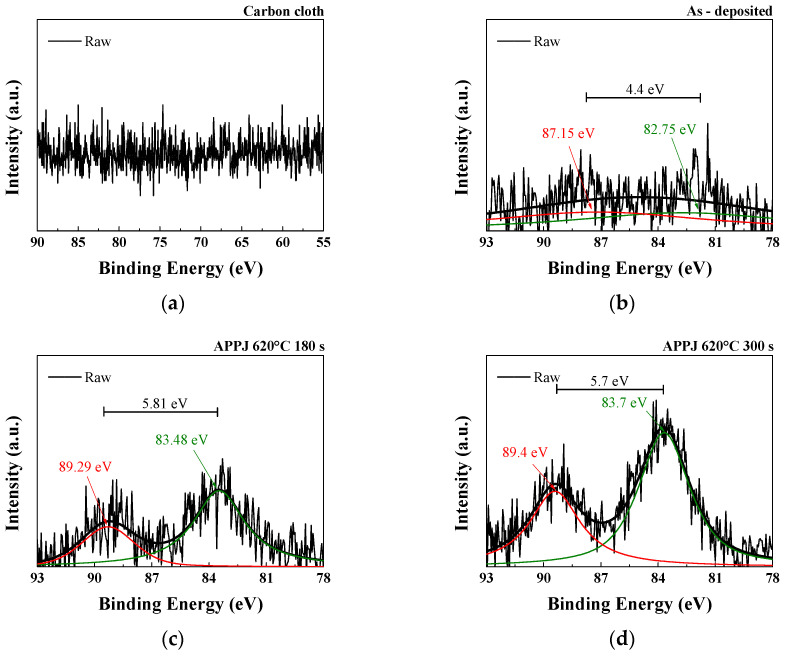
XPS Mn3s spectra of (**a**) carbon cloth and (**b**) untreated pastes on carbon cloth and APPJ-treated samples for (**c**) 180 s and (**d**) 300 s.

As shown in Figure 9, the Mn3s spectrum exhibits a doublet pattern, with a high-spin state (2p3/2) observed at a lower binding energy and a low-spin state (2p1/2) observed at a higher binding energy. According to the conventional linear equation ($V_{Mn}=7.875-0.893\Delta E_{3s}$), the average Mn valences are 3.946 for the as-deposited sample, 2.597 for that treated with APPJ for 180 s, and 2.785 for that treated with APPJ for 300 s, as shown in Table 3 [41]. According to the analysis results of O1s, the APPJ treatment caused the oxidation state adjustment of the manganese oxide surface, in which the Mn–O–H bonding decreased and the lattice oxygen (Mn–O–Mn) increased. These changes indicate that the degree of oxidation of manganese ions changed from a high oxidation state (Mn^4+^) to a low oxidation state (Mn^3+^). Samples treated with an APPJ for 300 s showed a higher average valence than that of samples treated with an APPJ for 180 s, suggesting that manganese was in a higher oxidation state, having lost more electrons and formed more bonds with oxygen atoms. This indicates a relatively higher degree of oxidation.

**Figure 10:**
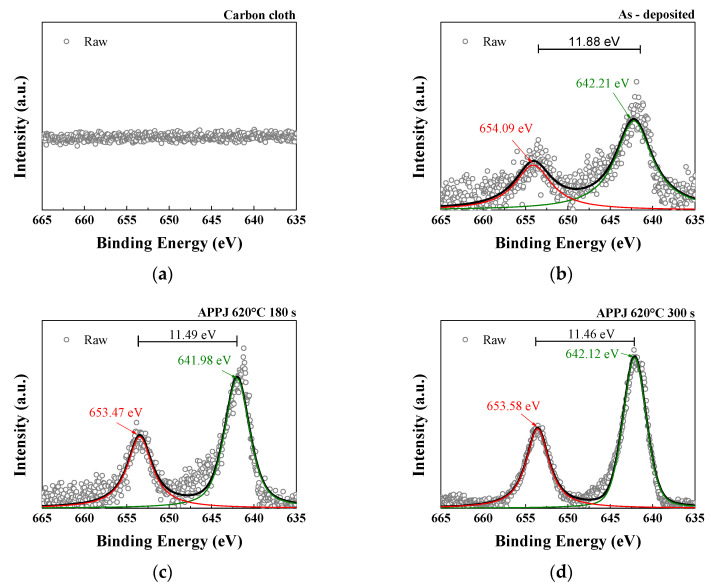
XPS Mn2p spectra of (**a**) carbon cloth and (**b**) untreated pastes on carbon cloth and APPJ-treated samples for (**c**) 180 s and (**d**) 300 s.

As shown in Figure 10, the Mn 2p core-level spectrum contains two distinct peaks: $Mn\;2p_{3/2}$ and $Mn\;2p_{1/2}$. The binding energy values of these two peaks can be used to calculate the spin-orbital splitting value. The $Mn\;2p_{3/2}$ binding energy in the sample falls within the range of binding energies observed in Mn_2_O_3_ (641.6 eV) and MnO_2_ (642.6 eV). This finding suggests the concurrent presence of both ${Mn}^{3+}$ and ${Mn}^{4+}$ species in the sample [42].

### 3.4. XRD

Figure S1 presents the XRD patterns of carbon cloth, as-deposited pastes containing rGO-LiCl-Mn(NO_3_)_2_·4H_2_O, and the pastes treated with APPJ. In XRD analysis, before and after the APPJ treatment, there are no distinct peaks observed for the lithium manganese oxide material. Only the diffraction peak of the carbon cloth appears. This suggests that the rGO-LiMnOx material may possess a structure with low crystallinity.

### 3.5. CV of HSCs

**Figure 11:**
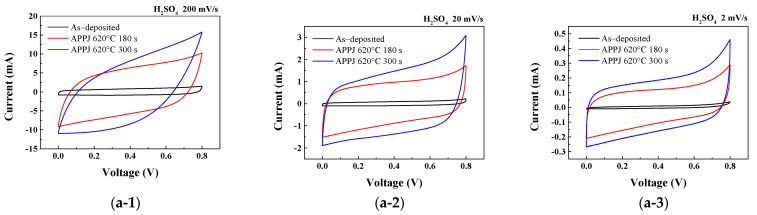

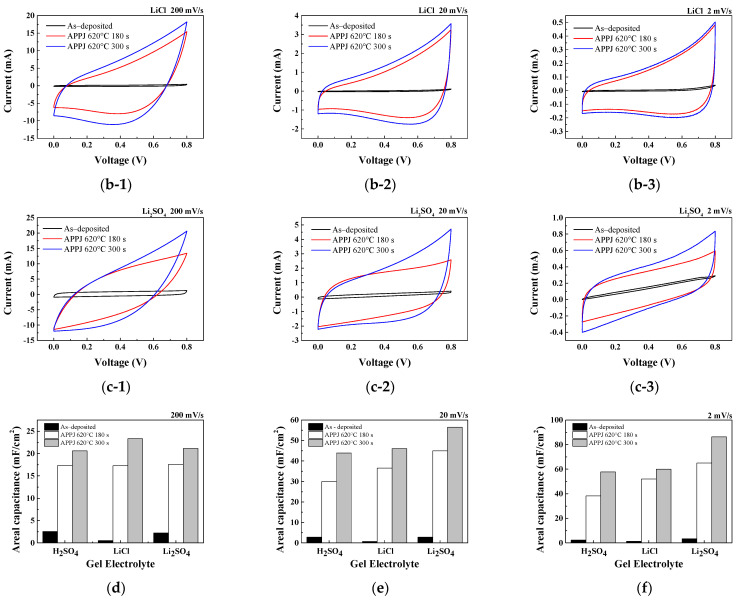
CV curves obtained for HSCs using 1 M (**a**) H_2_SO_4_, (**b**) LiCl, and (**c**) Li_2_SO_4_ gel electrolytes. Comparison of areal capacitance at different potential scan rates of (**d**) 200 mV/s, (**e**) 20 mV/s, and (**f**) 2 mV/s. (**a-1**–**a-3**) are CV curves of H_2_SO_4_ gel-electrolyte SCs with scan rates of 200 mV/s, 20 mV/s, and 2 mV/s, respectively. (**b-1**–**b-3**) are CV curves of LiCl gel-electrolyte SCs with scan rates of 200 mV/s, 20 mV/s, and 2 mV/s, respectively. (**c-1**–**c-3**) are CV curves of Li_2_SO_4_ gel-electrolyte SCs with scan rates of 200 mV/s, 20 mV/s, and 2 mV/s, respectively.

CV measurements provided insights into the electrochemical behavior and capacitance performance of the HSCs. As shown in Figure 11, the CV curves obtained for each HSC under different gel electrolytes and fabrication processes were analyzed and compared. The areal capacitance, C_A_, is calculated as

(1) $$C_{A}=\frac{1}{Av\Delta V}\int_{V_{a}}^{V_{c}}I\left(V\right)dV$$

through integrating the current (I) with respect to the potential (V) over the potential range and dividing it by the potential scan rate (*v*) and effective electrode area (A) [43]. Table 4, Table 5 and Table 6 sequentially represent the areal capacitance of HSCs fabricated using H_2_SO_4_, LiCl, and Li_2_SO_4_ gel electrolytes at different scan rates. The areal capacitance increased with decreasing scan rate and improved significantly after APPJ treatment. The best areal capacitance was achieved when using Li_2_SO_4_ gel electrolyte, and the largest area under the CV curve was observed with APPJ treatment at 620 °C for 300 s. When scanned at a rate of 2 mV/s, it results in an areal capacitance of 86.42 mF/cm^2^. The increase in capacitance at lower scan rates is attributed to two main factors. First, at lower scan rates, ions are given sufficient time to engage in the redox reaction, thus contributing to PC. Second, ions have more time to adsorb/desorb on the electrode surface, thus contributing to EDLC. Figure S2 and Table S1 (please see the Supplementary Material/File for more information) show the capacitance contribution ratio calculated using the Trasatti analysis method [44]. Trasatti analysis indicates that, compared to H_2_SO_4_, LiCl and Li_2_SO_4_ exhibit a higher proportion of PC. In LiCl and Li_2_SO_4_, lithium ions with high migration rates easily intercalate into the electrode material during charge/discharge cycles, thereby increasing the effective surface area for electrochemical reactions. This leads to a larger interface between the electrode material and the electrolyte, facilitating more sites for the Faradaic reactions involving lithium ion intercalation/extraction. As a result, the PC generated via the insertion of lithium ions combines with the EDLC, resulting in a significant overall increase in capacitance. In addition, the enhanced capacitance and energy density following surface modification primarily arises from the improved wettability of the electrode material, leading to an increased number of accessible sites for the formation of the electric double layer (EDL) [45]. Overall, the Li_2_SO_4_ gel electrolyte HSC treated with an APPJ at 620 °C for 300 s shows the highest areal capacitance, demonstrating the effectiveness of these factors in enhancing the electrochemical performance of the system. Table S3 (please see the Supplementary Material/File for more information) compares the electrochemical performance of different rGO-LiMnO_x_/rGO-MnO_x_-based supercapacitors.

### 3.6. GCD of HSCs

**Figure 12:**
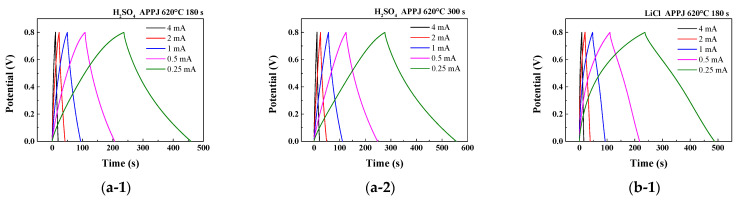

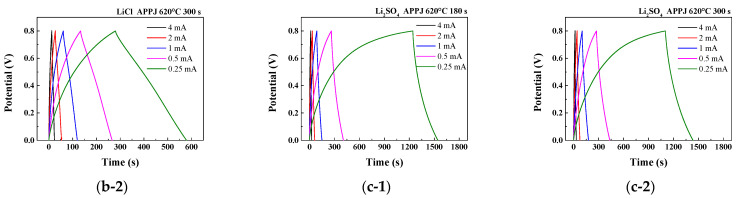
GCD curves obtained for HSCs using 1 M (**a**) H_2_SO_4_, (**b**) LiCl, and (**c**) Li_2_SO_4_ gel electrolytes under five constant currents: 4 mA, 2 mA, 1 mA, 0.5 mA, and 0.25 mA. (**a-1**,**a-2**) are plots obtained forH_2_SO_4_ gel-electrolyte SCs treated by APPJ for 300 s and 180 s, respectively. (**b-1**,**b-2**) are plots obtained for LiCl gel-electrolyte SCs treated by APPJ for 300 s and 180 s, respectively. (**c-1**,**c-2**) are plots obtained for Li_2_SO_4_ gel-electrolyte SCs treated by APPJ for 300 s and 180 s, respectively.

The electrochemical performance of HSCs was evaluated using GCD analysis under five constant currents. The areal capacitance, C_A_, is calculated as

(2) $$C_{A}=\frac{{2I}_{d}T_{d}}{A\Delta V}$$

where $I_{d}$ is the charging/discharging current; $T_{d}$, the discharging time; A, the electrode area; and ΔV, the potential scan window [44]. The discharge curve can be segmented into three regions: an abrupt potential drop caused by the HSC’s internal resistance, a rapid potential decrease attributed to the EDLC effect, and a gradual potential decay region resulting from PC behavior [46,47]. As shown in Figure 12, the GCD curves obtained for HSCs using different gel electrolytes and fabrication processes were analyzed and compared. A charge–discharge curve with an isosceles triangle shape is characteristic of EDLC. However, the figures suggest that the charge storage mechanism involves surface redox reactions rather than pure EDLC [48]. This confirms the results obtained from the CV analysis. As shown in Figure 12(c-1,c-2), when using the Li_2_SO_4_ gel electrolyte, the charging curve exhibits a smaller slope, indicating a more significant presence of oxidation–reduction reactions and slower reaction rates during charging. Table 7, Table 8 and Table 9 present the areal capacitance values obtained from the calculations using GCD results. The HSC using the Li_2_SO_4_ gel electrolyte with APPJ treatment at 620 °C for 300 s exhibits the highest performance, with an areal capacitance of 69.16 mF/cm^2^ when discharged at a current of 0.25 mA. A lower charging/discharging current implies that the HSC’s charging/discharging process is slower. This allows reactions to occur for a longer duration on the electrode surface, resulting in more charge transfer and electrochemical reactions. The ions in the electrolyte can undergo more complete adsorption and desorption on the electrode surface, thereby increasing the available surface area of the electrode and resulting in an increased calculated areal capacitance. These results as well as those of the previous CV analysis indicate that the Li_2_SO_4_-gel-electrolyte HSC exhibits better performance compared to that of HSCs with the other two gel electrolytes; however, it does not provide an optimal areal capacitance at a scan rate of 200 mV/s. This suggests that the HSC with the Li_2_SO_4_ gel electrolyte may have a lower ion conductivity, leading to incomplete reactions at higher scan rates and resulting in a smaller areal capacitance [49].

### 3.7. Ragone Plot

**Figure 13:**
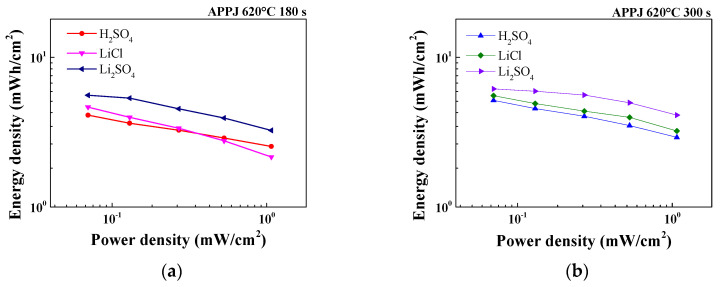
Comparison of Ragone plots for H_2_SO_4_, LiCl, and Li_2_SO_4_ gel electrolyte HSCs treated with APPJ for (**a**) 180 s and (**b**) 300 s.

The Ragone plot shown in Figure 13 was analyzed based on the GCD measurement results. The energy density and power density were respectively calculated using Equations (3) and (4) as

(3) $$E_{A}=\frac{C_{A}\times \Delta V^{2}}{7.2}\;$$

(4) $$P_{A}=\frac{{3.6\times E}_{A}}{T}$$

where $E_{A}$ is the energy density; $C_{A}$, the areal capacitance calculated using the GCD method; ΔV, the potential scan window; $P_{A}$, the power density; and T, the discharging time [50]. As shown in Table 10, the HSC using the Li_2_SO_4_ gel electrolyte with APPJ treatment at 620 °C for 300 s has the highest performance, with an energy density of 6.15 μWh/cm^2^ when discharged at a current of 0.25 mA. Under a discharging current of 4 mA, the highest power density of 1.07 mW/cm^2^ was achieved.

### 3.8. Stability of HSCs

**Figure 14:**
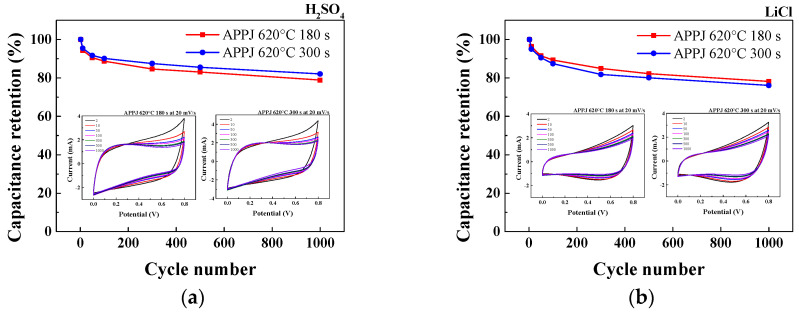

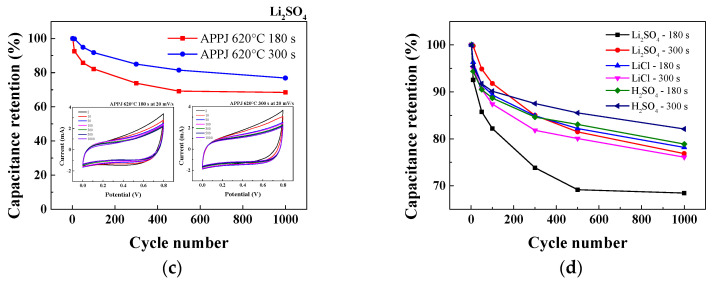
Cycling CV test with a potential scan rate of 20 mV/s using 1 M (**a**) H_2_SO_4_, (**b**) LiCl, and (**c**) Li_2_SO_4_ gel electrolytes. (**d**) Capacitance retention for 1000-cycle CV stability test.

The stability of HSCs was evaluated through a 1000-cycle CV test with a potential scan rate of 20 mV/s. As shown in Figure 14, the HSC with H_2_SO_4_ gel electrolyte and APPJ treatment at 620 °C for 300 s exhibits the highest capacitance retention rate of 82.1% after 1000 cycles. With LiCl and Li_2_SO_4_ gel electrolytes, the capacitance retention rate was approximately 70% or higher. The rate of decay decreased and then leveled off as the number of cycles increased. As shown in Video S1, the fabricated HSC was charged to power an LED and thereby demonstrate its energy storage capability.

### 3.9. EIS of HSCs

From the EIS analysis in Figure S3 (please see the Supplementary Material/File for more information), it can be observed that the slopes of the impedance curves for the three types of gel electrolytes are close to 45 degrees, indicating an approximation to PC behavior. Among them, the slope of the H_2_SO_4_ curve is larger compared to the other two, suggesting a higher contribution of EDLC, consistent with the results of Trasatti analysis. Additionally, lithium sulfate exhibits smaller values of Rs and Rct, corresponding to a higher electron propagation speed and enhanced redox reaction compared to the other electrolytes [51,52].

## 4. Conclusions

We demonstrate that it is feasible to fabricate electrodes of HSCs through screen-printing pastes containing rGO and LiCl-Mn(NO_3_)_2_·4H_2_O onto a carbon cloth substrate, followed by treatment with a nitrogen APPJ. H_2_SO_4_, LiCl, and Li_2_SO_4_ gel electrolyte HSCs were successfully fabricated using APPJ-processed rGO-LiMnO_x_ electrodes. Electrochemical testing revealed that the areal capacitance of the HSCs increased after APPJ treatment, and both energy density and cycling stability improved with longer APPJ treatment times. Among those HSCs, the one with the Li_2_SO_4_ gel electrolyte exhibited the highest areal capacitance and energy density. However, it showed relatively poor stability in stability testing. By contrast, the HSC with the H_2_SO_4_ gel electrolyte exhibited a lower areal capacitance and energy density but better capacitance retention rate.

## Figures and Tables

**Table 1:** XPS analysis of the C1s spectra in Figure 6, providing the atomic ratio of carbon bonding states.

	C–C (at%)	C–O (at%)	C=O (at%)	O–C=O (at%)
Carbon cloth	100	-	-	-
As-deposited	32.28	46.59	10.33	10.80
APPJ-180 s	52.88	24.75	14.08	8.29
APPJ-300 s	61.02	23.43	8.08	7.47

**Table 2:** XPS analysis of the O1s spectra in Figure 7, providing the atomic ratio of carbon bonding states.

	Lattice Oxygen Mn–O–Mn (at%)	Mn–O–H (at%)	C–O (at%)	C=O (at%)
Carbon cloth	-	-	100	-
As-deposited	6.73	50.76	42.49	0.02
APPJ-180 s	23.41	47.55	3.60	25.43
APPJ-300 s	60.11	28.49	6.35	5.05

**Table 3:** Average valence of Mn based on the XPS analysis of the Mn 3s spectra shown in Figure 9.

	Mn–O–H (at%)	C–O (at%)	C=O (at%)
$\Delta E_{3s}$ (eV)	4.4	5.81	5.7
Average valence of Mn	3.946	2.597	2.785

**Table 4:** Areal capacitance of HSCs using H_2_SO_4_ gel electrolyte, calculated based on CV results.

$\mathrm{Areal}\;\mathrm{Capacitance}\;(mF/cm^{2}$)			
APPJ Treatment	Potential Scan Rate (mV/s)		
	200 mV/s	20 mV/s	2 mV/s
As-deposited	2.55	2.81	2.32
APPJ-180 s	17.32	29.92	38.26
APPJ-300 s	20.60	43.91	57.76

**Table 5:** Areal capacitance of HSCs using LiCl gel electrolyte, calculated based on CV results.

$\mathrm{Areal}\;\mathrm{Capacitance}\;(mF/cm^{2}$)			
APPJ Treatment	Potential Scan Rate (mV/s)		
	200 mV/s	20 mV/s	2 mV/s
As-deposited	0.53	0.74	1.34
APPJ-180 s	17.26	36.52	51.96
APPJ-300 s	23.33	46.04	59.95

**Table 6:** Areal capacitance of HSCs using Li_2_SO_4_ gel electrolyte, calculated based on CV results.

$\mathrm{Areal}\;\mathrm{Capacitance}\;(mF/cm^{2}$)			
APPJ Treatment	Potential Scan Rate (mV/s)		
	200 mV/s	20 mV/s	2 mV/s
As-deposited	2.22	2.81	3.37
APPJ-180 s	17.60	44.92	65.13
APPJ-300 s	21.15	56.47	86.42

**Table 7:** Areal capacitance of HSCs using H_2_SO_4_ gel electrolyte, calculated based on GCD results.

$\mathrm{Areal}\;\mathrm{Capacitance}\;(mF/cm^{2}$)					
APPJ Treatment	Discharging Current				
	4 mA	2 mA	1 mA	0.5 mA	0.25 mA
APPJ-180 s	28.56	32.46	36.62	40.86	46.28
APPJ-300 s	32.81	39.42	45.42	51.23	58.09

**Table 8:** Areal capacitance of HSCs using LiCl gel electrolyte, calculated based on GCD results.

$\mathrm{Areal}\;\mathrm{Capacitance}\;(mF/cm^{2}$)					
APPJ Treatment	Discharging Current				
	4 mA	2 mA	1 mA	0.5 mA	0.25 mA
APPJ-180 s	24.28	31.19	37.85	44.62	52.28
APPJ-300 s	36.27	44.70	49.16	55.22	62.27

**Table 9:** Areal capacitance of HSCs using Li_2_SO_4_ gel electrolyte, calculated based on GCD results.

$\mathrm{Areal}\;\mathrm{Capacitance}\;(mF/cm^{2}$)					
APPJ Treatment	Discharging Current				
	4 mA	2 mA	1 mA	0.5 mA	0.25 mA
APPJ-180 s	36.55	44.16	51.00	60.05	62.76
APPJ-300 s	46.25	55.96	62.96	66.82	69.16

**Table 10:** Energy density of HSCs using H_2_SO_4_, LiCl, and Li_2_SO_4_ gel electrolytes, calculated based on GCD results.

$\mathrm{Energy}\;\mathrm{Density}\;(\mu Wh/cm^{2}$)						
		Discharging Current				
		4 mA	2 mA	1 mA	0.5 mA	0.25 mA
H_2_SO_4_	APPJ-180 s	2.54	2.89	3.26	3.63	4.11
	APPJ-300 s	2.92	3.50	4.04	4.55	5.16
LiCl	APPJ-180 s	2.16	2.77	3.36	3.97	4.65
	APPJ-300 s	3.22	3.97	4.37	4.91	5.54
Li_2_SO_4_	APPJ-180 s	3.25	3.93	4.53	5.34	5.58
	APPJ-300 s	4.11	4.97	5.60	5.94	6.15

## Data Availability

Not applicable.

## Supplementary Materials

The following supporting information can be downloaded at: https://www.mdpi.com/article/10.3390/mi14091701/s1, Figure S1. Trasatti plots generated for HSCs using 1 M (**a**) H_2_SO_4_, (**b**) LiCl, and (**c**) Li_2_SO_4_ gel electrolytes. Plots of (**a-1**,**b-1**,**c-1**) $C_{A}$ vs. $1/v^{0.5}$ and (**a-2**,**b-2**,**c-2**) $1/C_{A}$ vs. $v^{0.5}$. (**d**) Capacitance contributions ratio of PC/EDLC.; Figure S2. Trasatti plots generated for HSCs using 1 M (**a**) H_2_SO_4_, (**b**) LiCl, and (**c**) Li_2_SO_4_ gel electrolytes. Plots of (**a-1**,**b-1**,**c-1**) C_A vs. 1/$v^{0.5}$ and (**a-2**,**b-2**,**c-2**) 1/C_A vs. $v^{0.5}$. (**d**) Capacitance contributions ratio of PC/EDLC; Figure S3. Comparison of EIS results for H_2_SO_4_, LiCl, and Li_2_SO_4_ gel electrolyte HSCs treated with APPJ for 300 s; Table S1. Capacitance contributions of HSCs.; Table S2. Advantages of the work compared to reported electrolyte supercapacitors; Table S3. Comparison of electrochemical properties of rGO-LiMnOx/rGO-MnOx based supercapacitors [4,27,53,54,55,56,57,58]; Video S1: Demonstration of lighting up LED with a charged HSC.
