# Supplementary material for: Ultrafast Fabrication of H2SO4, LiCl, and Li2SO4 Gel Electrolyte Supercapacitors with Reduced Graphene Oxide (rGO)-LiMnOx Electrodes Processed Using Atmospheric-Pressure Plasma Jet
Source: Micromachines (Basel). 2023 Aug 30;14(9):1701. doi: 10.3390/mi14091701 (PMC10535643; doi:10.3390/mi14091701)
Supplement: Supplementary file 1 [file micromachines-14-01701-s001.zip › micromachines-2563068 - supplementary - final version.pdf]

micromachines

MDPI

Supplementary Material

Ultrafast Fabrication of H_2SO_4, LiCl, and Li_2SO_4 Gel Electrolyte Supercapacitors with Reduced Graphene Oxide (rGO)-$LiMnO_x$ Electrodes Processed Using Atmospheric-Pressure Plasma Jet

Pei-Ling Lan [1,2], I-Chih Ni [3], Chih-I Wu [3,4], Cheng-Che Hsu [5], I-Chun Cheng [3] and Jian-Zhang Chen [1,2,4,*]

[1] Graduate Institute of Applied Mechanics, National Taiwan University, Taipei City 10617, Taiwan; r11543037@ntu.edu.tw
[2] Advanced Research Center for Green Materials Science and Technology, National Taiwan University, Taipei City 10617, Taiwan
[3] Department of Electrical Engineering, Graduate Institute of Photonics and Optoelectronics, National Taiwan University, Taipei City 10617, Taiwan; ichihni@ntu.edu.tw (I.-C.N.); chihiwu@ntu.edu.tw (C.-I.W.); iccheng@ntu.edu.tw (I.-C.C.)
[4] Graduate School of Advanced Technology, National Taiwan University, Taipei City 10617, Taiwan
[5] Department of Chemical Engineering, National Taiwan University, Taipei City 10617, Taiwan; chsu@ntu.edu.tw
* Correspondence: jchen@ntu.edu.tw; Tel.: +886-2-3366-5694

Supplementary Information

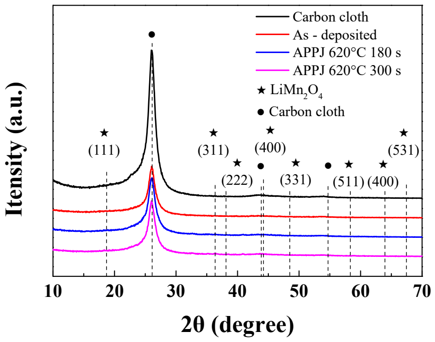

Figure S1. XRD patterns of rGO-$LiMnO_x$ on carbon cloth.

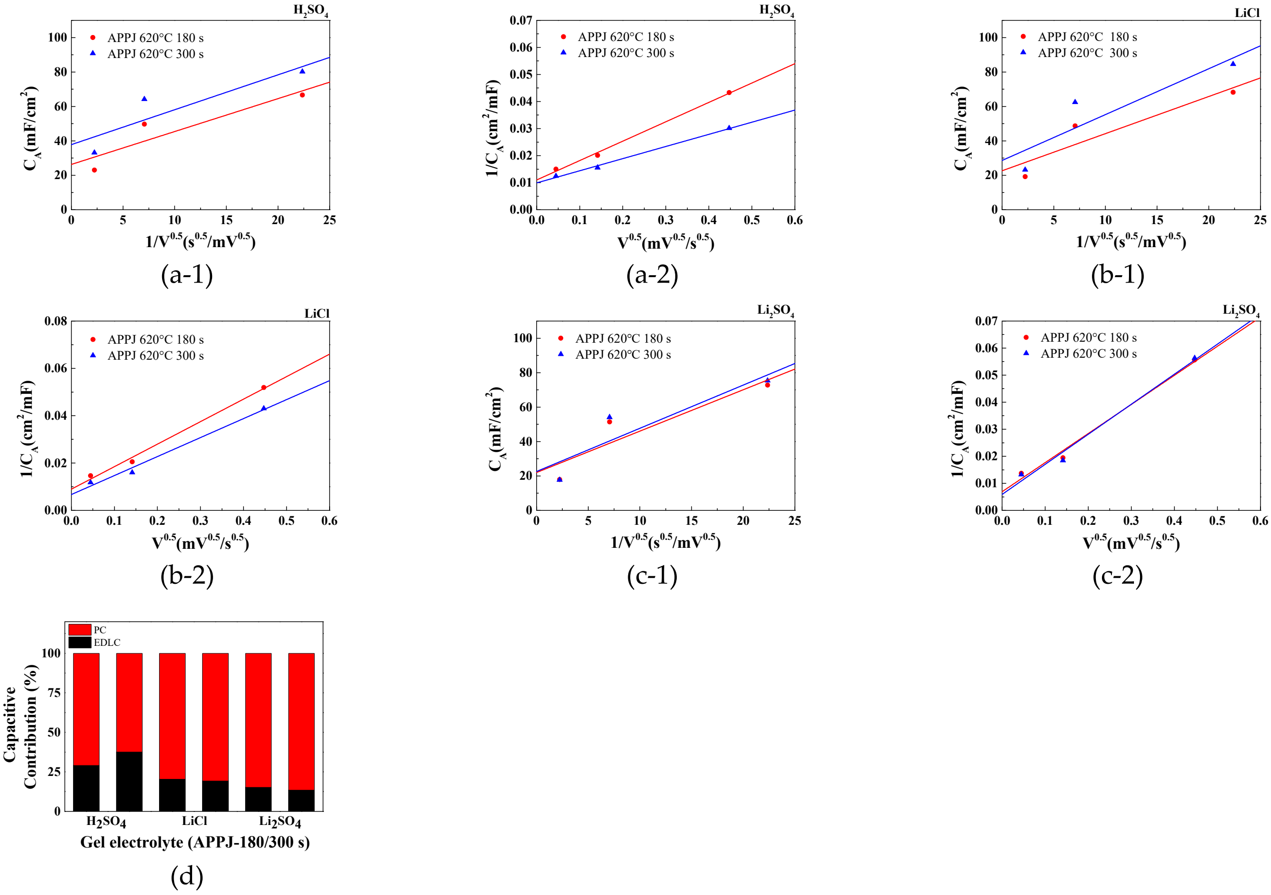

Figure S2. Trasatti plots generated for HSCs using 1 M (**a**) H_2SO_4, (**b**) LiCl, and (**c**) Li_2SO_4 gel electrolytes. Plots of (**a-1**, **b-1**, **c-1**) C_A vs. $1/v^{0.5}$ and (**a-2**, **b-2**, **c-2**) $1/C_A$ vs. $v^{0.5}$. (**d**) Capacitance contributions ratio of PC/EDLC.

Table S1. Capacitance contributions of HSCs.

		C_{total} (mF/cm²)	C_{out} (mF/cm²)	C_{in} (mF/cm²)	Capacitive Contribution (EDLC:PC) (%)
H_2SO_4	APPJ - 180 s	90.74	26.32	64.42	29.0:71.0
	APPJ - 300 s	100.70	37.82	62.88	37.6:62.4
LiCl	APPJ - 180 s	111.48	22.65	88.83	20.3:79.7
	APPJ - 300 s	149.03	28.64	120.39	19.2:80.8
Li_2SO_4	APPJ - 180 s	144.51	22.03	122.48	15.2:84.8
	APPJ - 300 s	169.20	22.62	146.58	13.4:86.6

Table S2. Advantages of the work compared to reported electrolyte supercapacitors.

Advantages	**Description**
Novel material combination	By screen-printing pastes containing rGO and LiCl-Mn(NO3)2·4H2O onto carbon cloth substrate with nitrogen APPJ processing, a novel material combination with superior performance is introduced.
Potential advantages of choosing different gel electrolytes	The interaction between gel electrolyte and electrode materials leads to higher energy storage and faster energy release, resulting in superior energy density

	and power density compared to reported supercapacitors.
Stability	Nitrogen APPJ treatment enhances interface stability between materials, thereby extending the operational lifespan of the supercapacitor.

Table S3. Comparison of electrochemical properties of rGO-LiMnO$_x$/ rGO-MnO$_x$ based supercapacitors.

Electrode materials	Electrolyte	Performance	Cycling stability	Reference
Carbon cloth/ rGO-LiMnOx	PVA/ 1M H_2SO_4	57.76 mF/cm^2	82.10% after 20 mV/s for 1000 cycles	This work
Carbon cloth/ rGO-LiMnO$_x$	PVA/ 1M LiCl	59.95 mF/cm^2	76.09% after 20 mV/s for 1000 cycles	This work
Carbon cloth/ rGO-LiMnO$_x$	PVA/ 1M Li_2SO_4	86.42 mF/cm^2	76.86% after 20 mV/s for 100 cycles	This work
Aluminum foil/ rGO-$LiMnO_2$	1M LiPF6	185.60 mAh g^{-1}	80.97% after 100 cycles	[1]
Aluminum foil/ $LiMnO_2$	1M LiPF6	140.8 mAh g^{-1}	53.53% after 100 cycles	[1]
RGO wrapped $LiMn_2O_4$ nano-rods	1M LiPF6	143.5 mAh g^{-1}	97% after 50 cycles	[2]
Carbon cloth/ rGO-MnO$_x$	PVA/ 1M H_2SO_4	57.01 mF/cm^2	84.41% after 200 mV/s for 10,000 cycles	[3]
Carbon cloth/ $LiMn_2O_4$	1M Li_2SO_4	78.46 mC cm^{-2}	104.53% after 20 mV/s for 1000 cycles	[4]
Nickel foam/ rGO-Mn_3O_4	1M Na_2SO_4	457 F g^{-1}	91.60% after 5000 cycles	[5]
Nickel foam/ rGO-MnO$_x$	1M Na_2SO_4	214 F g^{-1}	N/A	[6]
defect induced rGO-MnO$_x$	1M Na_2SO_4	264 F g^{-1}	153% after 10000 cycles	[7]
Nickel foam/ rGO-MnO$_x$/Mn_3O_4	0.5M Na_2SO_4	442 F g^{-1}	N/A	[8]

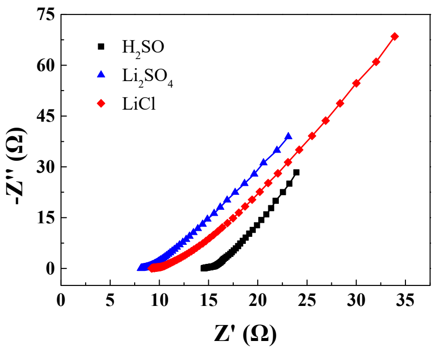

Figure S3. Comparison of EIS results for H_2SO_4, LiCl, and Li_2SO_4 gel electrolyte HSCs treated with APPJ for 300 s.

References

1. Tian, Y.; Qiu, Y.; Liu, Z.; Wei, X.; Cao, H. $LiMnO_2$@rGO nanocomposites for high-performance lithium-ion battery cathodes. *Nanotechnology*. **2021**, *32*, 015402, https://doi.org/10.1088/1361–6528/abb48c.
2. He, J.; Chen, Y.; Li, P.; Fu, F.; Liu, J.; Wang, Z. Facile fabrication of GRO wrapped $LiMn_2O_4$ nanorods as a cathode with enhanced specific capacity. *RSC Advances* **2015**, *5*, 80063–80068. https://doi.org/10.1039/C5RA09783E.
3. Shah, H.U.; Wang, F.; Javed, M.S.; Shaheen, N.; Saleem, M.; Li, Y. Hydrothermal synthesis of reduced graphene oxide-Mn_3O_4 nanocomposite as an efficient electrode materials for supercapacitors. *Ceramics International* **2018**, *44*, 3580–3584, https://doi.org/10.1016/j.ceramint.2017.11.062.
4. Zhao, X.; Yang, H.; Hou, Y.; Gbologah, L.; Zhu, L.; Wang, Y. Systematically controlled synthesis of rGO/nano-MnO_x materials for supercapacitors. *Progress in Natural Science: Materials International* **2019**, *29*, 504–510, https://doi.org/10.1016/j.pnsc.2019.09.003.
5. Prakash, D.; Manivannan, S. Defect induced RGO-MnO_x hybrid electrodes for supercapacitor applications. *AIP Conference Proceedings* **2019**, *2115*, https://doi.org/10.1063/1.5113420.
6. Mokhtarnejad, M.; Ribeiro, E.L.; Mukherjee, D.; Khomami, B. 3D printed interdigitated supercapacitor using reduced graphene oxide-$MnO_{(x)}$ /$Mn_{(3)}O_{(4)}$ based electrodes. *RSC Adv.* **2022**, *12*, 17321–17329, https://doi.org/10.1039/d2ra02009b.

Disclaimer/Publisher's Note: The statements, opinions and data contained in all publications are solely those of the individual author(s) and contributor(s) and not of MDPI and/or the editor(s). MDPI and/or the editor(s) disclaim responsibility for any injury to people or property resulting from any ideas, methods, instructions or products referred to in the content.
